# AmpC β-Lactamase-Producing Microorganisms in South American Hospitals: A Meta-Regression Analysis, Meta-Analysis, and Review of Prevalence

**DOI:** 10.3390/tropicalmed10100280

**Published:** 2025-09-29

**Authors:** Valmir Nascimento Rastely-Junior, Hosanea Santos Nascimento Rocha, Mitermayer Galvão Reis

**Affiliations:** 1 Faculdade de Medicina da Bahia, Universidade Federal da Bahia (UFBA), Salvador 40026-010, Brazil; valmir.j.rastely@gmail.com; 2 Departamento de Medicina, Escola Bahiana de Medicina e Saúde Pública (EBMSP), Salvador 40290-000, Brazil; 3 Instituto Gonçalo Moniz (IGM), Fundação Oswaldo Cruz (Fiocruz), Salvador 40296-710, Brazil

**Keywords:** AmpC β-lactamases, hospital isolates, South America, antimicrobial resistance, meta-analysis, systematic review, prevalence, enterobacter

## Abstract

AmpC β-lactamases are class C enzymes that hydrolyze penicillins, cephalosporins, and monobactams. The WHO recently classified third-generation cephalosporin-resistant and carbapenem-resistant Enterobacterales as critical pathogens. We conducted a systematic review and meta-analysis to evaluate AmpC prevalence in hospital isolates across South America. We searched PubMed/MEDLINE, SciELO, and Google Scholar. We included 69 observational studies that phenotypically or genotypically identified AmpC producers. A random-effects generalized linear mixed model with logit transformation estimated pooled prevalence; heterogeneity and moderators were explored through subgroup analyses and meta-regression. Seventy studies, including 48,801 isolates, were eligible. AmpC β-lactamases were detected in 11.7% of isolates (95% CI 11.4–12.0), with extreme heterogeneity (I^2^ ≈ 97%). Enterobacter species showed the highest prevalence (~46%), whereas *Escherichia* spp. had the lowest (~4.5%) prevalence of AmpC positivity within each genus. Meta-regression indicated that studies focusing on a single genus reported higher prevalence and that including pediatric patients was associated with a lower prevalence of AmpC-positive microorganisms among isolates. Quality of evidence was rated low due to inconsistency, moderate risk of bias, and indirectness of data. AmpC producers are entrenched in South American hospitals, and species-aware surveillance and harmonized detection are critical to guide empiric therapy and antimicrobial stewardship.

## 1. Introduction

Antimicrobial resistance (AMR) is one of the biggest threats to global public health in the 21st century. It undermines decades of medical progress and puts the foundations of modern medicine at risk [1]. There are many ways that bacteria have learned to avoid antimicrobial agents, but the production of β-lactamase enzymes by Gram-negative pathogens is one of the main reasons why they are resistant to the β-lactam class of antibiotics [2]. These enzymes work by hydrolytically breaking the amide bond in the four-atom β-lactam ring, which makes the antibiotic molecule inactive [3]. While considerable research and clinical focus have been directed towards specific classes of β-lactamases, notably extended-spectrum β-lactamases (ESBLs), other equally significant enzymes have been relatively underexplored in numerous areas. AmpC β-lactamases constitute a unique and particularly formidable resistance mechanism, characterized by their distinct biochemical properties, intricate genetic regulation, and the substantial diagnostic and therapeutic challenges they pose in clinical contexts. AmpC β-lactamases are very good at breaking down a lot of β-lactam agents, such as most penicillins, monobactams like aztreonam, and, most importantly, first-, second-, and third-generation cephalosporins like cefotaxime, ceftazidime, and ceftriaxone [4].

The global crisis of AMR is not evenly spread out. Its causes and effects are often made worse by social and economic factors, which puts a disproportionate burden on countries with low and middle incomes [1]. The rise of drug-resistant pathogens is especially harmful for South America, which has a wide range of healthcare systems and populations. To find out what public health actions are most important, we need to know the details of this regional crisis. AMR is seen as a big threat in the area, but the scientific and public health response has mostly been based on the dramatic and well-documented rise in ESBLs. This may have made it easier to miss other, less well-known resistance mechanisms like AmpC [4]. The wealth of detailed and fairly recent epidemiological data on ESBLs stands in stark contrast to the thin, fragmented, and mostly out-of-date information on AmpC β-lactamases.

This fragmented and outdated dataset is fundamentally inadequate for guiding evidence-based public health policy, clinical practice, and infection control across a continent as large and diverse as South America. It is impossible to accurately assess the true public health burden of AmpC-mediated resistance and justify or direct such resources effectively without a consolidated, contemporary overview. Policymakers cannot determine whether AmpC prevalence is a low-level, stable issue or a rapidly emerging crisis that is simply going unmeasured [5].

This uncertainty has direct consequences for the patient’s bedside. The development of effective empirical antibiotic prescribing guidelines relies on accurate and up-to-date local and regional antibiograms [6]. In the absence of data, particularly data addressing AmpC prevalence, clinicians managing patients with severe Gram-negative infections must make judgments based on insufficient knowledge. This may result in the improper utilization of third-generation cephalosporins or β-lactam/β-lactamase inhibitor combos, leading to therapeutic failure, heightened morbidity, and extended hospitalizations [7].

This systematic review seeks to systematically identify, critically evaluate, and synthesize the existing evidence about the prevalence of AmpC β-lactamases in clinical isolates from hospital environments throughout South America.

## 2. Materials and Methods

### 2.1. Register and Guidelines

This systematic review and meta-analysis followed PRISMA 2020 statement guidelines [8] and was prospectively registered in PROSPERO (CRD420251084285). The study commenced on 2 July 2025 and was completed on 12 August 2025.

### 2.2. Eligibility Criteria

Observational studies conducted in hospitals in South America that identified AmpC-type β-lactamase-producing bacteria by phenotypic and genotypic methods were included. Studies with patients of any age, including mixed populations (inpatient and outpatient), were accepted, provided the data were predominantly related to the hospital setting or allowed for an estimation of prevalence in this context. The outcome of interest was AmpC prevalence, including studies providing indirect data (total number of isolates and number of positive isolates). There were no language or time restrictions. We excluded non-original studies (such as reviews, editorials, or letters), environmental investigations, case reports, in vitro models, and exclusively outpatient studies.

### 2.3. Information Sources

Studies were searched without language or time restrictions in MEDLINE via Pubmed, SciELO, and Google Scholar. The strategy used a sensitive string combining South American countries, hospital terms, and AmpC synonyms, adapted to each database’s syntax. In Google Scholar, there was no limit on the number of results. The search was complemented by citation tracking, reference lists, gray literature (theses, conference proceedings), and contact with experts. All references were organized in Zotero and deduplicated in Rayyan AI before screening. We considered including LILACS, but it was excluded because it yielded no unique eligible studies beyond those found in the combination of final chosen databases.

### 2.4. Search Strategy

Construction of the search string—A sensitive string was developed combining South American countries, hospital-related terms, and AmpC synonyms. The search string used in PubMed was as follows:

(Argentina OR Bolivia OR Brazil OR Chile OR Colombia OR Ecuador OR Guyana OR Paraguay OR Peru OR Suriname OR Uruguay OR Venezuela OR “South America*” OR “South America”[Mesh]) AND (“critical care*” OR “critical care*”[mesh] OR “ICU” OR “intensive care*” OR “Intensive Care Units”[Mesh] OR “neurocritic*” OR “critically ill” OR “Critical Illness*” OR “Critical Illness”[mesh] OR “hospital*” OR “Hospital Units”[Mesh] OR “Hospitals”[Mesh] OR “Hospital Departments”[Mesh] OR “emergenc*” OR “Emergency Medical Services”[Mesh] OR “nosocomial”) AND (“AmpC*” OR “pAmpC*” OR “cAmpC*” OR “C β-lactamase” OR “C beta-lactamase” OR “ampicillinase Cs” OR “ampicillinase C”).

The search strategies used in other databases are included in Supplementary Materials.

### 2.5. Study Selection Process

All retrieved records were imported into Zotero and Rayyan, and duplicates were removed. Two reviewers then independently screened titles/abstracts and full texts. A third, independent screen was performed with Claude Sonnet 3.7, which classified records against pre-specified eligibility criteria using the labels include/exclude/uncertain. Disagreements about the three screening procedures were reviewed by two authors, and final inclusion decisions were made by human consensus.

### 2.6. Data Extraction

Initially, two reviewers independently extracted the data. Secondly, a third extraction was produced and compared with the first ones using assistance from Claude Sonnet 4.7. After that, all fields and any disagreements were reviewed by two authors and final decisions were made by human consensus, using a standardized spreadsheet with a data dictionary, piloted beforehand to calibrate interpretations. For each study, the following were recorded: identification (first author, year of publication, and study period), country, study design, definition and hospital setting, sample size, age range (mean or median and range), type of diagnostic test for AmpC and eligibility criteria applied, isolated bacterial species and genera, year of sampling, total number of microorganisms isolated, and total number of AmpC-positive isolates. We classified isolates as AmpC producers when the source study provided (a) phenotypic confirmation using recognized approaches—typically cefoxitin-based screening followed by inhibitor-based confirmation (e.g., boronic acid or cloxacillin), AmpC disk tests, or equivalent confirmatory assays—or (b) genotypic confirmation by PCR and/or sequencing of ampC determinants (e.g., blaCMY, blaDHA, blaFOX, blaACC, blaMOX, blaEBC/ACT/MIR). When both phenotypic and genotypic results were available, genotypic confirmation took precedence. This process minimized extraction errors and ensured independence between reviewers.

### 2.7. Risk-of-Bias Assessment

Risk of bias in the included studies was independently assessed by two reviewers using the “JBI Critical Appraisal Checklists for Prevalence Study” for each study design [8]. In each study, reviewers classified risk as low, moderate, or high; disagreements were discussed until consensus was reached.

### 2.8. Data Synthesis

All analyses were conducted in R version 4.3.2 (2023-10-31 ucrt) using the *meta* (v8.2-0), *metafor* (v4.8-0), *mice* (v3.17.0), *clubSandwich* (v0.6.1), *ggplot2* (v3.5.2) and *dplyr* (v1.1.4) packages.

Prevalence estimates were pooled using a binomial–normal generalized linear mixed model (GLMM) fitted on the logit scale [9,10]. For each outcome (number of AmpC-positive isolates divided by total isolates), we used meta::metaprop() (R package *meta*, v8.2-0) with method = “GLMM” and summary measure sm = “PLOGIT” to fit a random-effects GLMM with a logistic link and maximum likelihood estimator for between-study variance τ^2^. Confidence intervals for the pooled proportion and τ^2^ were obtained via the Hartung–Knapp adjustment. The GLMM does not require continuity corrections; however, if the model failed to converge (e.g., due to sparse data or quasi-separation), we used a Freeman–Tukey double-arcsine transformation (sm = “PFT”) with restricted maximum likelihood (REML) for τ^2^ as a fallback [11]. Heterogeneity was quantified by the Q statistic, τ^2^, and I^2^. When at least ten studies were available, publication bias was assessed using funnel plots and Egger’s test. A sensitivity analysis excluded studies with 0% or 100% observed prevalence.

Univariable meta-regression models were fitted to explore heterogeneity. For each moderator (year of sampling, country, diagnostic test type, inclusion of outpatients, inclusion of children/adolescents, sample origin, focused bacterial group, and detection objective), a random-intercept GLMM was fitted using metafor::rma.glmm() (package *metafor*, v v4.8-0) with the measure set to PLO (logit of the proportion) and maximum likelihood estimation [11]. Categorical moderators were coded with the most common category as reference, and levels represented by <5 studies were collapsed to “Other”. The year variable was centered, and the risk-of-bias score was centered for interpretation. Model fit and the reduction in τ^2^ relative to the intercept-only model (pseudo-R^2^) were reported. For each model, we attempted several approximation methods (CM, CM.EL, CM.AL, UM.FS) and optimisers (BFGS, Nelder–Mead, nlminb) with 20 000 iterations and relative tolerance 10^−10^; if no GLMM converged, we fitted a REML random-effects model on the logit-transformed proportions with a continuity correction of 0.5 added to all cells. Robust standard errors were obtained via the HC3 method using *clubSandwich* [12,13].

For the multivariable meta-regression, we simultaneously evaluated three prespecified moderators: inclusion of children/adolescents (yes/no), the risk-of-bias score (centered), and whether the study focused on a specific bacterial group (specific vs. various/any). Because these covariates were missing for some studies, we used multiple imputation (*mice*, v3.17.0) under the missing-at-random assumption. The imputation dataset included the logit effect size (yi), its variance (vi), study identifiers, total isolates, total AmpC-positive isolates, and the three moderators. Missing values were imputed using logistic regression (logreg) or polytomous regression (polyreg) for categorical variables and predictive mean matching (pmm) for the continuous risk-of-bias score. We generated 20 imputed datasets (m = 20), each with 20 iterations (maxit = 20), and used the default predictor matrix. Each imputed dataset was analyzed with the GLMM described above; if a GLMM failed, the REML fallback was used. Regression coefficients and their standard errors were combined across imputations using Rubin’s rules via mice::pool(), and pooled confidence intervals were calculated. Between-study heterogeneity (τ^2^) and pseudo-R^2^ were averaged across imputations. Diagnostics included inspection of imputation distributions, fraction of missing information, influence analysis, sensitivity to exclusion of zero- and full-prevalence studies, and collinearity diagnostics (Pearson correlations, Cramér’s V, variance inflation factors, condition indices) [13,14]. A reproducible R code and all analysis scripts are available from the authors on request.

### 2.9. Certainty of Evidence Assessment (GRADE)

The certainty of the evidence was assessed using the GRADE approach [14], applied to the primary outcome of prevalence. Each pooled estimate was rated as high, moderate, low, or very low based on five domains: methodological limitations of the included studies, inconsistency across results, imprecision of estimates, indirectness relative to the review question, and potential publication bias.

## 3. Results

A systematic search across Medline, Scielo, and Google Scholar yielded over 15,000 records, but rigorous screening and duplication checks left 336 articles for full-text appraisal and 69 studies meeting the inclusion criteria, as shown in Figure S1. These studies collectively span nearly thirty years, indicating that AmpC β-lactamase producers have been present in South American hospitals since the late 1990s [15,16,17,18,19] and continue to be detected into the 2020s [20,21,22,23,24,25,26,27,28]. Surveillance intensified in the mid-2000s through sequential cross-sectional series and prospective cohorts, showing persistent circulation across institutions [29,30,31,32,33]. More recent point-prevalence studies and multicentric surveys from 2013 onward confirm that AmpC remains a contemporary challenge [21,34,35,36,37].

Table 1 shows that the evidence base includes a wide range of methods. Cross-sectional studies are predominant, indicating dependence on routine laboratory surveillance and facilitating prevalence comparisons across hospitals and time [29,38,39,40,41]. Although fewer in number, prospective and retrospective cohort studies elucidate temporal dynamics and correlations with clinical outcomes [16,21,25,42,43,44,45]. Molecular studies and outbreak investigations elucidate genetic mechanisms and transmission pathways [22,24,31,46,47]. Study durations varied from short outbreaks lasting a few weeks to extensive surveillance spanning nearly a decade [27,41,48,49], demonstrating that AmpC detection occurs regardless of follow-up duration.

Brazil and Colombia provide most of the data [39,45,60,64,65,68,69,70,71,77,81,84], with additions from large series from Argentina and Chile. Reports from Peru, Ecuador, Bolivia, Venezuela, and Uruguay also add to the data [31,54,59,75,82]. The settings examined are similarly diverse, encompassing adult and neonatal/pediatric ICUs and specialized units—such as oncology and transplant services—and extending to general wards and emergency departments. This variety shows that AmpC producers are not limited to high-risk units; they can be found throughout the hospital. While numerous studies concentrated on adults, a notable subset examined pediatric patients [56,63,72,76,83], especially in neonatal and pediatric ICUs, demonstrating that children are also impacted.

The majority of researchers evaluated clinical infections, corresponding with the clinical relevance of AmpC enzymes, whereas a lesser number examined both colonization and infection [29,35,53,61,62,66,74,78,80]. Only one study concentrated exclusively on colonization, underscoring a deficiency in the comprehension of asymptomatic reservoirs [43]. There were different ways to find things: phenotypic tests were the most common, but they were often used with genotypic tests to make them more accurate. The coexistence of multi-species and species-specific studies allowed for both broad and targeted insights. Stratification by species is important in meta-analyses. Most included articles were judged to be at low risk of bias using the JBI tool, indicating a generally robust evidence base, although a few studies exhibited moderate risk due to methodological limitations.

### 3.1. Meta-Analysis Results

The meta-analysis included 69 studies and 48,801 isolates of Gram-negative bacteria from South American hospitals. Using a random-effects GLMM with a logit transformation and maximum-likelihood estimator, the pooled prevalence of AmpC β-lactamase production across all microorganisms was 11.7% (95% CI 11.4–12.0%; τ^2^ = 0 on the logit scale; I^2^ = 97.2%; Q = 2429.55, *p* < 0.001; 95% prediction interval [PI] 11.4–12.0%), as shown in Table 2. Egger’s test showed no evidence of small-study bias (intercept ≈ 0; *p* = 0.779). The high I^2^ may reflect marked between-study and between-country variability (Figure S2). When studies reporting 0% or 100% prevalence were excluded, the pooled prevalence decreased modestly to 10.0% (95% CI 9.8–10.3%), with τ^2^ still 0 and I^2^ remaining high, indicating that heterogeneity was not driven solely by extreme findings.

Subgroup analyses by genus revealed substantial differences in intra-genus prevalences. Among Enterobacterales, *Enterobacter* spp. had the highest intra-genus pooled prevalence, at 46.0% (95% CI 43.7–48.4%; τ^2^ = 0; I^2^ = 30.9%; Q = 41.96, *p* = 0.057; PI 43.6–48.5%; k = 30). Although heterogeneity was moderate, Egger’s test indicated mild small-study effects (*p* = 0.037). *Salmonella* spp. (k = 7) showed a pooled prevalence of 28.5% (95% CI 21.2–37.0%; τ^2^ = 0; I^2^ = 0%; PI 19.6–39.3%); Egger’s test was not performed because of the small number of studies. *Morganella* spp. (k = 18) had a prevalence of 20.2% (95% CI 14.8–27.0%; τ^2^ = 0; I^2^ = 0%; PI 14.5–27.6%), with no evidence of small-study bias (*p* = 0.499). *Serratia* spp. (k = 21) showed 20.0% (95% CI 17.2–23.0%; τ^2^ = 0; I^2^ = 0%; PI 17.1–23.2%; Egger *p* = 0.852). *Citrobacter* spp. (k = 19) had a prevalence of 18.9% (95% CI 14.0–25.2%; τ^2^ = 0; I^2^ = 0%; PI 13.7–25.6%; Egger *p* = 0.086). *Klebsiella* spp. (k = 42) yielded 7.05% (95% CI 6.60–7.53%; τ^2^ = 0; I^2^ = 78.7%; Q = 192.15, *p* < 0.001; PI 6.6–7.6%), with significant small-study effects (*p* = 0.005). *Proteus* spp. (k = 27) showed 5.23% (95% CI 3.87–7.03%; τ^2^ = 0; I^2^ = 0%; PI 3.8–7.1%; Egger *p* = 0.584). *Escherichia* spp. (k = 39) had the lowest prevalence at 4.48% (95% CI 4.03–4.98%; τ^2^ = 0; I^2^ = 93.4%; Q = 575.38, *p* < 0.001; PI 4.0–5.0%), and Egger’s test suggested small-study bias (*p* = 0.028).

Among non-Enterobacterales, *Acinetobacter* spp. (k = 12) had a prevalence of 16.7% (95% CI 14.8–18.7%; τ^2^ = 0; I^2^ = 0%; PI 14.6–19.0%; Egger *p* = 0.046), while *Pseudomonas* spp. (k = 28) showed 19.3% (95% CI 18.0–20.7%; τ^2^ = 0; I^2^ = 83.4%; Q = 162.31, *p* < 0.001; PI 18.0–20.7%; Egger *p* = 0.249). Sensitivity analyses excluding 0%/100% studies yielded similar or modestly lower prevalence estimates for most genera, indicating that extreme proportions were not driving the pooled results.

### 3.2. Meta-Regression Results

**Figure 1:**
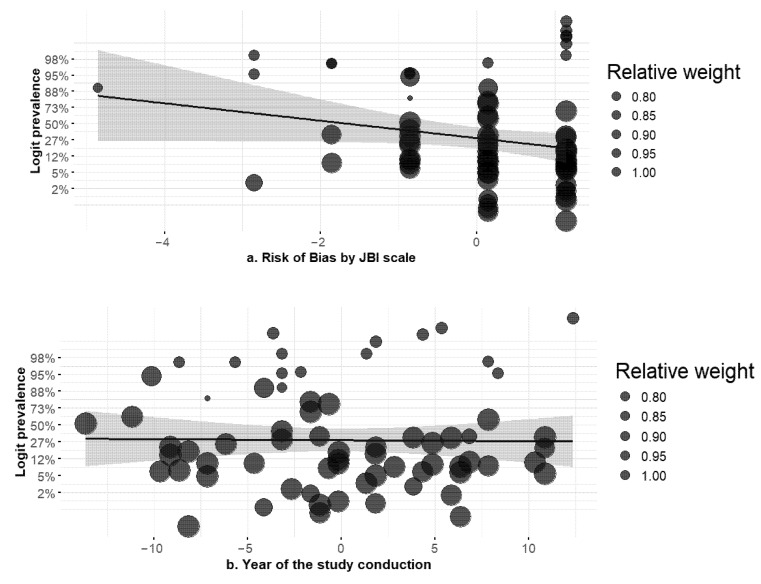
Meta-regression of AmpC β-lactamase prevalence in South American hospital isolates by (**a**) JBI risk-of-bias score and (**b**) study year. Abbreviations: AmpC: AmpC β-lactamase; JBI: Joanna Briggs Institute; CI: confidence interval; REML: restricted maximum likelihood; PI: prediction interval; β: meta-regression slope; k: number of studies. Notes. Bubbles are proportional to inverse-variance study weights from a random-effects meta-analysis. Lines show meta-regression fits with 95% CI shading; tests of moderators (β, 95% CI, *p*) are reported in each panel. Proportions were modeled on the logit scale and back-transformed to percentages for *y*-axis labeling; therefore, the axis is titled “Prevalence (%)” even though modeling used logit units. Panel (**a**) uses the JBI overall risk-of-bias score; if scores were centered, “centered on the mean” is stated explicitly; otherwise, raw scale values are displayed (e.g., 0–9). Panel (**b**) uses the mid-year of data collection for each study; when only publication year was available, the publication year was used as a proxy (the count of such cases is stated in Methods).

The univariable meta-regressions evaluated each moderator separately (Table S1). Years of data collection showed no evidence of a temporal trend (Figure 1). Each additional year was associated with β = −0.03 (95% CI −0.14 to 0.08) and an odds ratio (OR) of 0.97 (95% CI 0.87–1.08); the *p*-value was 0.566 and the pseudo-R^2^ was 0%. This indicates that AmpC prevalence did not systematically increase or decrease over time, and nearly all variability remained unexplained.

**Figure 2:**
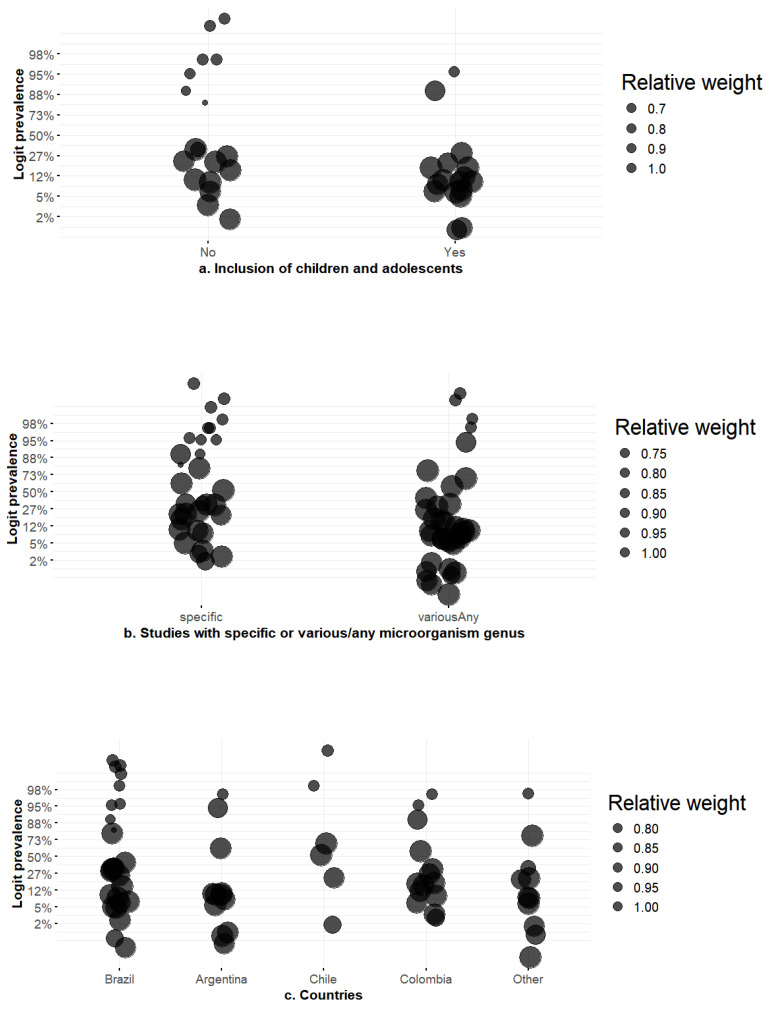
Subgroup distribution of AmpC β-lactamase prevalence in South American hospitals by (**a**) pediatric inclusion, (**b**) organism scope, (**c**) country. Footnote: Abbreviations: AmpC: AmpC β-lactamase; CI: confidence interval; RE: random effects; REML: restricted maximum likelihood; k: number of studies; n: number of isolates. Notes. Each point represents a study-level estimate of the proportion of AmpC-positive isolates; bubble size is proportional to the inverse-variance weight from the RE model. Proportions were modeled on the logit scale and back-transformed to percentages for the *y*-axis; thus, the axis reads Prevalence (%), although fitting used logits. For each subgroup, the pooled prevalence with 95% CI (diamond/bar) and the number of contributing studies (k) are displayed. The test of subgroup differences (Cochran’s Q_between and *p*) or the meta-regression slope (β), 95% CI, p is reported inside each panel. In panel (**c**), “Other countries” includes those with small numbers of studies (e.g., Peru, Uruguay, Venezuela, Ecuador, Bolivia). We added a 95% prediction interval behind each pooled diamond when heterogeneity within subgroups remained high.

As shown in Figure 2, country effects were suggestive but not statistically robust: compared with Brazil, studies from Argentina had β = −1.67 (95% CI −3.60 to 0.26), OR = 0.19 (95% CI 0.03–1.30), and p = 0.090; Chile showed β = 1.68 (95% CI −0.75 to 4.10), OR = 5.34 (95% CI 0.47–60.46), *p* = 0.176; Colombia had β = −0.70 (95% CI −2.55 to 1.15), OR = 0.50 (95% CI 0.08–3.14), *p* = 0.457. The omnibus test (QM = 8.989, *p* = 0.061) and pseudo-R^2^ of 6.6% suggest some geographic heterogeneity, but with wide confidence intervals and substantial residual heterogeneity (τ^2^ ≈ 75.4, I^2^ ≈ 99.3%).

The type of AmpC test did not account for variability. Compared with purely phenotypic testing, using both phenotypic and genotypic methods had β = 0.39 (95% CI −1.21 to 1.99), OR = 1.47 (95% CI 0.30–7.29), *p* = 0.634, and using genotypic methods alone had β = 1.07 (95% CI −0.70 to 2.83), OR = 2.90 (95% CI 0.50–17.01), *p* = 0.237. Including outpatients likewise had no significant effect (β = −0.98, 95% CI −2.81 to 0.86; OR = 0.38 [95% CI 0.06–2.36], *p* = 0.297). Methodological quality (risk of bias) showed a marginal association: each one-point increase in the JBI score corresponded to β = −0.53 (95% CI −1.10 to 0.04), OR = 0.59 (95% CI 0.33–1.04), and *p* = 0.070, with a pseudo-R^2^ of 4.1%. Bubble plots showed a slight downward slope. The sample origin (infection vs. colonization) showed no effect: comparing “both infection and colonization” versus infection had β = −0.25 (95% CI −2.13 to 1.64), OR = 0.78 (95% CI 0.12–5.14), *p* = 0.798, and comparing colonization alone versus infection had β = −0.17 (95% CI −6.08 to 5.73), OR = 0.84 (95% CI effectively undefined), *p* = 0.954.

Inclusion of children and adolescents was a statistically significant moderator. Studies that included pediatric populations had β = −1.77 (95% CI −3.31 to −0.22), OR = 0.17 (95% CI 0.04–0.80), and *p* = 0.025, explaining 9.7% of the heterogeneity; prevalence was roughly one-third of that in adult-only studies. Taxonomic focus emerged as a strong determinant: studies focused on a single bacterial genus or species had much higher prevalence than those screening a variety of genera. The contrast of “various/any” versus “specific” yielded β = −1.99 (95% CI −3.29 to −0.68), corresponding to OR = 0.14 (95% CI 0.04–0.51), *p* = 0.003, and a pseudo-R^2^ of 11.1%. This means that the odds of detecting an AmpC producer were reduced by ~86% when the study sampled multiple genera. The detection objective showed a borderline effect: when AmpC was a secondary rather than primary outcome, β = −1.42 (95% CI −3.00 to 0.16), OR = 0.24 (95% CI 0.05–1.17), *p* = 0.078, and pseudo-R^2^ = 2.3%.

In the multivariable meta-regression (Table S2), three moderators were entered simultaneously. Focusing on a variety of genera remained a strong independent predictor of lower prevalence, with β = −1.96 (95% CI −3.35 to −0.57), OR = 0.14 (95% CI 0.04–0.57), and *p* = 0.007. Inclusion of children/adolescents retained a large effect (β = −1.74, 95% CI −3.64 to 0.15; OR = 0.17 [95% CI 0.03–1.16]), but the *p*-value increased to 0.069, suggesting partial confounding with study focus. Study quality (risk of bias) was no longer significant (β = −0.04, 95% CI −0.70 to 0.62; OR = 0.96 [95% CI 0.50–1.85], *p* = 0.901). The multivariable model explained 19.5 % of between-study variance, yet residual heterogeneity remained extreme (τ^2^ ≈ 62.6, I^2^ ≈ 99.3%).

In summary, while most potential moderators—such as year of study, detection method, inclusion of outpatients, and sample origin—did not meaningfully explain the wide variation in AmpC β-lactamase prevalence across South American hospital studies, two factors consistently stood out. Studies targeting a single bacterial genus (often known high-risk groups) reported substantially higher prevalence than studies sampling multiple genera, and studies that included children or adolescents tended to find much lower prevalence. The findings indicate that variations in AmpC prevalence are primarily influenced by taxonomic emphasis and population age rather than by temporal trends, geographic disparities, or methodological factors, underscoring the necessity to meticulously evaluate study scope and demographics when comparing prevalence estimates.

## 4. Discussion

### 4.1. Principal Findings

A total of 69 studies encompassing 48,801 isolates were also eligible for meta-analysis. Using a random-effects generalized linear mixed model, we found that roughly one in ten isolates carried AmpC β-lactamase enzymes (pooled prevalence 11.7%, 95% CI 11.4–12.0%). The heterogeneity among studies was substantial (I^2^ = 97%), indicating significant variance in occurrence across different environments. Genus stratification indicated that AmpC producers were most prevalent on an intra-genus basis in *Enterobacter* spp. (~46%), moderate in *Serratia* spp. (~20%, almost no heterogeneity) and *Pseudomonas* spp. (~19%), appreciable in *Salmonella* spp. (~28%, few isolates), but much lower in *Klebsiella* spp. (~7%) and *Escherichia* spp. (~4.5%).

AmpC β-lactamase prevalence in South America appears elevated compared to most other regions. Europe and North America have some of the lowest documented AmpC rates globally (often only ~1–5% of Enterobacterales isolates) [85]. In contrast, parts of Asia and the Middle East show much greater AmpC burdens—certain local studies (for example, reports from India and Nepal) have observed AmpC prevalence rates exceeding 20–40% in clinical isolates. Africa’s experience seems more intermediate, with considerable variability by country; for instance, plasmid-mediated AmpC in *E. coli* has ranged from under 1% in some areas to nearly 15% in Egypt [86]. This places South America’s prevalence at an upper-middling level globally—lower than the highest rates seen in Asian hotspots, but significantly above the prevalence typically found in European or North American settings.

Addressing prevalence and geographic heterogeneity, the included studies indicate that AmpC β-lactamases are less frequent than ESBLs in South American hospital isolates, with global estimates for ESBLs typically at 10–40%. Clinically and phenotypically, AmpC differs from ESBLs by resistance to clavulanate and hydrolysis of cephamycins, and co-occurrence with ESBLs is reported—features that confound phenotypic algorithms and may inflate or obscure AmpC prevalence [87,88]. Regionally, the marked heterogeneity documented for ESBLs (e.g., shifting CTX-M variants) underscores the likelihood of locally variable AmpC epidemiology that current evidence cannot resolve [89]. Methodologically, underdetection is likely: widely used phenotypic screens have suboptimal sensitivity/specificity—especially for plasmid-mediated AmpC. CLSI lacks a standardized detection method. Multiplex PCR, while more reliable, is rarely routine, collectively biasing prevalence downward [4,87]. In parallel, South American hospitals face high burdens of ESBLs and carbapenemase producers, intensifying the clinical relevance of any unmeasured AmpC contribution and complicating interpretation of resistance phenotypes and empiric coverage decisions [90,91,92].

Finally, One Health linkages—documented across human, animal, and environmental interfaces—reinforce that hospital burdens are both recipients and amplifiers of broader transmission networks, further justifying coordinated interventions beyond the acute care setting [89].

### 4.2. Interpretation of Heterogeneity

Despite stratification and meta-regression, between-study variability remained very high. The taxonomic focus of studies consistently explained some heterogeneity: investigations targeting a single high-risk genus (e.g., *Enterobacter* spp.) reported far higher prevalence than those screening multiple genera. Multivariable meta-regression estimated that sampling a variety of genera reduced the odds of detecting an AmpC producer by roughly 86%. Population age also moderated prevalence: studies that included children and adolescents had markedly lower prevalence rates (odds ratio ≈ 0.17), possibly reflecting different exposure patterns, microbiota, or prescribing practices in pediatric wards. Other variables—such as year of conduction, country, detection method, inclusion of outpatients, and sample origin (infection vs. colonization cases)—did not significantly account for heterogeneity. The persistence of high between-study variance may reflect differences in species ecology and sampling frames, ward types (ICU vs. general), selective culturing, thresholds for screening, and case mix. Focusing on high-risk species can inflate prevalence, whereas broad screening across genera may dilute detection, underscoring the need for species-aware surveillance.

### 4.3. Implications for Policy and Surveillance

The high and heterogeneous burden of AmpC β-lactamases underscores the need for regional standardization of detection and reporting. South American health networks should develop unified phenotypic algorithms and build capacity for targeted molecular confirmation, ensuring that results are comparable across laboratories. Surveillance should integrate colonization and ambulatory settings to capture spill-over between hospitals and communities. Data sharing across countries will enable benchmarking of species-stratified prevalence and inform region-wide antibiograms. Public health authorities should incorporate AmpC reporting into national antimicrobial resistance plans, linking microbiology data with stewardship and infection control programs.

It is essential to recognize that the region possesses a robust policy framework and surveillance capacity for antimicrobial resistance—led by the PAHO/WHO and the ReLAVRA+ network. Yet hospital-level implementation of antimicrobial stewardship programs (ASPs) remains heterogeneous. Evidence syntheses report widespread adoption of national action plans and highlight ReLAVRA+ as the epidemiological backbone but also show that ASP maturity is concentrated in a few countries and that many institutions lack the infrastructure to monitor antimicrobial use and align prescribing with local microbiology [93,94,95]. Nevertheless, where well designed, ASPs are associated with lower antibiotic consumption and improved clinical outcomes, while strengthening pathogen detection and guiding de-escalation—strategies particularly relevant to reducing selective pressure from cephalosporins and carbapenems, implicated in the emergence of AmpC [96]. Recent advances include PAHO–GARDP partnerships to expand rational access to antimicrobials and build regional capacity, while feasible interventions such as multiprofessional education and prospective audit with feedback have demonstrated measurable impacts (e.g., shorter time to first dose in septic patients in Latin American ICUs) [97]. In sum, mitigating the burden of AmpC in the region requires translating intent into operational capacity—investing in multidisciplinary teams, monitoring technologies, and the continuous use of ReLAVRA+ data to inform local protocols and evaluate impact [98].

### 4.4. Clinical and Microbiological Implications

High-risk genera such as *Enterobacter* spp. account for nearly half of isolates tested, indicating that clinicians should remain vigilant when treating infections caused by these taxa. Species with intrinsic inducible ampC (*Enterobacter cloacae complex*, *Citrobacter freundii*, *Klebsiella aerogenes*) can initially appear susceptible to third-generation cephalosporins but become resistant after induction [4]. Clinically, misclassification of AmpC as ESBL risks inappropriate empiric therapy and failure given frequent multidrug resistance and cephamycin hydrolysis [88]. For inducible chromosomal AmpC in high-risk species, on-therapy derepression can raise β-lactam MICs and threaten clinical efficacy [99]. Quantitative data show substantial cefepime MIC shifts with derepression in *Enterobacter cloacae complex* [100]. Conversely, low-risk species (*Serratia marcescens*, *Providencia* spp., *Morganella morganii*) have a lower propensity for clinically significant derepression, permitting carbapenem-sparing regimens when susceptibility is confirmed [90]. Among alternatives, cefepime is supported by a meta-analysis of observational studies in bloodstream infection showing no significant mortality difference versus carbapenems; IDSA suggests cefepime for high-risk organisms when MIC ≤ 2 μg/mL. Important caveats include potential inoculum effect and diminished reliability with ESBL co-production [91,92,101]. Evidence for piperacillin–tazobactam is mixed and generally weaker, with lower reported susceptibility among AmpC producers [99]. For selected scenarios (e.g., uncomplicated urinary tract infection or step-down), non-β-lactam agents have observational support without increased mortality [102]. Finally, laboratory reporting strategies can materially alter carbapenem use, underscoring the need to pair species-aware advice with stewardship and reporting practices in a dedicated “Practice Implications” subsection [103]. Therapeutic considerations in this review are therefore framed as observational/indirect and should be interpreted as guidance rather than directives.

The rising antimicrobial resistance (AMR) among nosocomial Gram-negative pathogens increases the clinical significance of AmpC β-lactamase-producing Enterobacterales isolated from hospital settings in South America and highlights the necessity for strategies that transcend mere incremental adjustments to current medications [104]. Due to the limited pipeline and the prevalence of resistance mechanisms that compromise traditional β-lactams, strategies that leverage bacterial metal homeostasis should be prioritized [104,105]. The most important of these are “Trojan horse” siderophore–antibiotic conjugates (SACs), which steal iron transport to get around porin loss and efflux and deliver active drugs into the periplasm [104]. The siderophore–cephalosporin cefiderocol represents this paradigm; its catechol moiety facilitates active absorption, while its β-lactam core maintains efficacy against targets such as PBP3 and demonstrates stability against Class C β-lactamases pertinent to AmpC manufacturers [106,107,108,109]. However, resistance to the strategy itself is emerging via mutations or downregulation of siderophore receptors (e.g., cirA, fiu, piuA/piuD/pirA) and via co-expression of multiple β-lactamases with permeability defects, necessitating surveillance for receptor alterations alongside routine resistance testing [110]. Moreover, co-selection dynamics—where plasmids carry both siderophore and resistance determinants—suggest that iron-limited conditions selecting for metallophore systems may also enrich resistance traits, reinforcing the need for integrated stewardship and genomic surveillance [111].

Iron chelation can be used as an adjuvant to break up biofilms and make traditional antibiotics work better [112,113]. Metal mimics (e.g., gallium) can also be used to stop iron-dependent metabolism [114]. Inhibition of metallophore production pathways is another method [114,115,116,117]. Resistance will develop through alterations in siderophore–receptor interactions, efflux mechanisms, and co-/cross-resistance associated with metal exposure, necessitating monitoring and management that are sensitive to these processes [110,118].

### 4.5. Infection Prevention and Stewardship

The genus-specific risks observed in this review translate into actionable surveillance priorities. Hospitals should focus screening resources on ICUs, oncology/transplant wards and neonatal/pediatric units, particularly where *Enterobacter* or *Serratia* spp. predominate. Preventive strategies should bundle device stewardship and contact precautions during clusters and environmental hygiene audits with antimicrobial stewardship (AMS) protocols that flag likely AmpC-producing organisms. Regular review of AmpC trends integrated with antibiotic consumption metrics will help evaluate the impact of interventions and adjust empiric therapy. Because AmpC overexpression can be induced by third-generation cephalosporins [99], stewardship programs should promote carbapenem-sparing regimens (high-dose cefepime or β-lactam/β-lactamase inhibitor combinations) where appropriate, while reserving carbapenems for severe infections or high-inoculum states [99].

### 4.6. Limitations

The evidence is mainly observational and hospital-based, limiting generalizability to community settings. Methodological heterogeneity (assay types, breakpoints, sampling frames) and varied species mixtures may contribute to residual I^2^ despite subgrouping and meta-regression. In addition, most primary studies did not consistently distinguish chromosomally mediated, inducible AmpC from plasmid-mediated AmpC (pAmpC). Consequently, our prevalence estimates reflect combined AmpC mechanisms, which may contribute to heterogeneity and should be interpreted with caution when comparing across genera. Generalizability may be further constrained by geographic and genera imbalance: the dataset is dominated by Brazil and Colombia—the two most populous South American countries—and some genera (e.g., *Salmonella* spp.) were rarely studied, yielding imprecise estimates and limiting continent-wide inference. Because the review focuses on hospital isolates rather than community or general isolates, the organisms captured are more representative of hospital settings. Given the ecological nature of country-level subgrouping, we emphasize that unmeasured confounders such as hospital size and surveillance policies may influence prevalence estimates and cannot be fully accounted for in this meta-analysis. Long surveillance series may have included duplicate isolates, and colonization was under-represented since most studies focused on clinical infections. Although funnel and Egger tests showed no asymmetry, they may be underpowered under such extreme heterogeneity.

### 4.7. Certainty of Evidence (GRADE)

Using GRADE principles, we rated the certainty of the pooled prevalence as low to moderate. We downgraded for very serious inconsistency (I^2^ ≈ 97%) and indirectness. Imprecision affected less common genera. Risk of bias was generally low, but some studies had moderate risk due to incomplete reporting or convenience sampling. Publication bias was not detected, but tests were underpowered. Decisions based on these estimates should recognize that true prevalence may vary locally; however, the consistent detection of AmpC across decades and settings supports the conclusion that these enzymes are established in South American hospitals.

### 4.8. Research Priorities

Future studies should adopt prospective, specific designs with standardized diagnostic protocols and clear denominators to produce comparable prevalence estimates. Pediatric-focused research is needed to validate the lower prevalence observed in children and to assess neonatal risks. Time-series analyses could elucidate temporal trends and the impact of interventions such as pandemic-related changes. Evaluations of diagnostic algorithms and AMS bundles would provide evidence on effective strategies. Genomic epidemiology can differentiate plasmid-mediated versus chromosomal ampC, map transmission, and detect co-mechanisms with ESBLs or carbapenemases. Studies exploring colonization reservoirs and transition points (e.g., emergency departments, step-down units, outpatient clinics) will help identify entry points for intervention. Clinically, misclassification of AmpC as ESBL risks inappropriate empiric therapy and failure given frequent multidrug resistance and cephamycin hydrolysis [89]. Priorities are standardized surveillance that reports AmpC separately from ESBLs, combined phenotypic/genotypic detection, routine assessment of co-resistance, and country-level analyses to capture true geographic variation across South American hospital environments [74,89].

## 5. Conclusions

AmpC β-lactamase-producing organisms are entrenched in South American hospitals. Prevalence averages about one in ten isolates, but varies enormously across genera and settings, with *Enterobacter* spp. exhibiting the highest rates. Yet residual heterogeneity remained extreme; meta-regression showed that heterogeneity was not explained by calendar time or detection method but was influenced by taxonomic focus and population age, indicating that study design and case mix shape prevalence estimates. Implementing species-aware surveillance and stewardship programs is immediately actionable, while harmonized diagnostic methodologies and broader surveillance are needed to reduce uncertainty and monitor trends over time.

## Figures and Tables

**Table 1:** Characteristics of studies reporting AmpC β-lactamase prevalence in hospital isolates from South America.

Study	Country	Data Collection Period (Years)	Design	No. of Centers	Care Setting (s)	Pediatric Patients	Outpatients	Case Type (Colonization vs. Infection)	AmpC Test Type
Favier (2021) [20]	Argentina	2016–2018	Quasi-experimental before–after	1	ICU, plus hospital-wide intervention	No	No	Infection	Phenotypic
Mora Moreno (2021) [38]	Colombia	2018	Cross-sectional	1	Multiple wards (ICU, emergency, surgery, internal medicine, orthopedics, pediatric hospitalization, pediatric emergency, gynecology)	NR	NR	NR	Both
Martínez (2020) [39]	Colombia	2014–2016	Cross-sectional descriptive	4	Hospitalization, ICU, operating rooms	No	Yes	NR	Phenotypic
Beirão (2020) [50]	Brazil	2016–2017	Prospective multicenter surveillance	8	Hospital (various wards: intra-abdominal, respiratory, urinary tract infections)	NR	No	Infection	Genotypic
Tuon (2020) [51]	Brazil	2016–2017	Multicenter prospective surveillance	10	ICUs and non-ICU wards	NR	No	Infection	Genotypic
Varon (2019) [52]	Colombia	2012	Retrospective cross-sectional	1	ICU	No	No	NR	Phenotypic
Aravena (2019) [29]	Chile	2006–2011	Cross-sectional	1	Hospital wards (specific wards NR)	NR	NR	NR	Both
Morales (2018) [40]	Colombia	2012–2016	Cross-sectional laboratory	Multiple	Hospital wards (specific wards NR)	NR	NR	Infection	Genotypic
Gómez-González (2018) [53]	Colombia	2015	Retrospective observational	1	ICU	No	No	Both	Phenotypic
Cacci (2016) [42]	Brazil	2007–2008	Prospective cohort	1	ICU	NR	NR	NR	Phenotypic
Arango (2016) [41]	Colombia	2006–2014	Descriptive cross-sectional	1	(PICU), NICU, oncology, burn unit, General Hospitalization, emergency room, surgery, Hematopoietic Transplant Unit	Yes	No	Infection	Phenotypic
Cavalcanti (2015) [30]	Brazil	2008–2010	Observational, laboratory-based	3	ICU, cardiology unit, oncology unit, adult isolation facility	NR	NR	NR	Genotypic
de la Lastra (2014) [54]	Chile	2010–2014	Cross-sectional laboratory surveillance	2	Hospital wards (specific wards NR)	No	No	Infection	Phenotypic
Campana (2013) [55]	Brazil	2006	Cross-sectional laboratory surveillance	1	Hospital wards (specific wards NR)	NR	No	Infection	Both
Cejas (2012) [48]	Argentina	2009	Prospective multicenter surveillance	7	Hospital wards (specific wards NR)	NR	Yes	Infection	Both
Hoyos (2012) [56]	Colombia	2009–2011	Cross-sectional	1	Pediatric emergency, neonatology, NICU, general pediatric ward	Yes	No	Infection	Phenotypic
Nastro (2012) [57]	Argentina	2009–2010	Prospective cross-sectional laboratory	1	Hospital wards (specific wards NR)	NR	Yes	Infection	Both
Marcano (2011) [58]	Venezuela	2009–2010	Cross-sectional, multicenter laboratory surveillance	8	Multiple wards	NR	Yes	Both	Both
Santella (2011) [31]	Argentina	2004–2005	Retrospective outbreak	1	Hospital wards (specific wards NR)	No	No	Infection	Genotypic
Jure (2011) [59]	Argentina	2009	Cross-sectional laboratory surveillance	3	Hospital wards (specific wards NR)	NR	Yes	Infection	Both
Vasques (2011) [43]	Brazil	2007	Prospective cohort	1	Cardiac ICU	Yes	No	Colonization	Genotypic
Ribas (2007) [16]	Brazil	2000–2001	Prospective cross-sectional surveillance	1	Tertiary acute care hospital	Yes	No	Infection	Phenotypic
Cantarelli (2007) [60]	Brazil	NR	Cross-sectional laboratory-based investigation	1	Hospital wards (specific wards NR)	NR	NR	NR	Both
Pino (2007) [15]	Chile	1995–1998	Laboratory surveillance	Multiple	Hospital wards (specific wards NR)	NR	No	Infection	Phenotypic
Bertona (2005) [17]	Argentina	1999	Cross-sectional laboratory surveillance	1	Hospital wards (specific wards NR)	NR	No	Infection	Phenotypic
García Romero (2005) [61]	Colombia	2001–2002	Cross-sectional	1	Multiple wards (ICU, surgical ward, nephrology, emergency, internal medicine, coronary care unit, orthopedics, neurology)	No	No	Both	Phenotypic
Cezário (2004) [62]	Brazil	2001–2002	Cross-sectional prevalence surveys	1	Multiple wards (NICU, High-Risk Unit, Intermediate Care, etc.)	Yes	NR	Both	Phenotypic
Silva (2024) [21]	Brazil	2013–2018	Prospective cohort	1	Surgical and clinical wards, ICU, coronary unit, ER, outpatient hemodialysis clinic	No	NR	Infection	Phenotypic
Soto (2024) [28]	Chile	2021–2024	Cross-sectional laboratory-based of cohort	1	Multiple wards	NR	NR	NR	Both
Rodrigues (2024) [23]	Brazil	2017–2020	Cross-sectional	1	ICU and NICU	No	No	Both	Both
Arns (2024) [25]	Brazil	2020–2022	Prospective multicentric cohort	5	Hospital wards (specific wards NR)	No	No	Infection	Phenotypic
Mojica (2023) [63]	Multiple countries	2016–2017	Cross-sectional laboratory-based surveillance	Multiple	Hospital wards (specific wards NR)	Yes	NR	NR	Genotypic
Villanueva-Cotrina (2022) [26]	Peru	2021	Case series	1	Specialized cancer center (inpatient, outpatient, emergency)	Yes	Yes	Infection	Phenotypic
Lee (2021) [64]	Brazil	2010–2014	Cross-sectional	12	Hospital wards (specific wards NR)	NR	NR	Infection	Genotypic
Ferreira (2021) [44]	Brazil	2012–2017	Cohort	1	Cancer hospital (inpatient wards, ICU, clinical/surgical wards)	No	No	Infection	Phenotypic
Perez (2020) [65]	Brazil	2016– 2017	Cross-sectional	1	Hospital wards (specific wards NR)	NR	No	Infection	Phenotypic
Papa-Ezdra (2020) [66]	Uruguay	2017	Case series	2	Hospital A (ICU, general ward), Hospital B (primary care clinic)	No	Yes	Both	Genotypic
Nocua-Báez (2017) [67]	Colombia	2014–2015	Cross-sectional	9	Emergency department and inpatient wards (adults, UTI)	No	NR	Infection	Both
Pavez (2016) [68]	Brazil	2005–2009	Laboratory-based	3	Hospital wards (specific wards NR)	NR	NR	NR	Both
Rocha (2016) [45]	Brazil	2012	Prospective cross-sectional laboratory	7	Hospital wards (specific wards NR)	Yes	NR	NR	Both
Martins (2014) [69]	Brazil	2008–2009	Laboratory-based surveillance	5	General hospitals (with ICU, emergency, outpatient)	NR	NR	Infection	Both
Leal (2013) [70]	Colombia	2011–2012	Analytical case–control	9	Hospital, mainly emergency services	No	No	Infection	Both
Zavascki (2012) [46]	Brazil	2008	Case series (Outbreak investigation)	1	ICU	Yes	No	Both	Both
Fehlberg (2012) [19]	Multiple countries	2000–2002	Comparative cross-sectional	2	Hospital wards (specific wards NR)	No	No	Infection	Genotypic
Nogueira-Miranda (2012) [71]	Brazil	2005–2008	Cross-sectional laboratory-based evaluation	1	Hospital wards (specific wards NR)	NR	NR	NR	Phenotypic
Cuzon (2011) [32]	Colombia	2006–2010	Cross-sectional laboratory surveillance	Multiple	ICU, general wards	NR	NR	Infection	Genotypic
Picão (2009) [72]	Brazil	2005	Retrospective survey	1	ICU, emergency room, pediatric oncology unit, bone marrow transplant, hemodialysis, surgery	Yes	NR	Infection	Phenotypic
Lincopan (2008) [73]	Brazil	2007	Case report	1	Surgical ward, transplant unit,	No	No	Infection	Both
Dias (2008) [74]	Brazil	2001	Retrospective cohort laboratory investigation	1	Hospital wards (specific wards NR)	NR	No	Both	Both
Sader (2005) [75]	Multiple countries	2000–2004	Multicenter laboratory surveillance	93	ICU	NR	No	Infection	Phenotypic
Casellas (2003) [76]	Argentina	2001–2002	Multicenter cross-sectional	17	Hospital wards (specific wards NR)	Yes	NR	NR	Phenotypic
Quinteros (2003) [18]	Argentina	2000	Multicenter cross-sectional survey	17	Hospital wards (specific wards NR)	NR	NR	NR	Both
Soria-Segarra (2020) [37]	Ecuador	2016	Observational prospective cohort	7	ICU	No	NR	NR	Phenotypic
Bartoloni (2016) [35]	Bolivia	2010–2014	Prospective surveillance	1	Hospital wards (specific wards NR)	Yes	Yes	Both	Both
Kiffer (2005) [77]	Brazil	2003	Multicenter laboratory surveillance	20	ICU, neutropenic units, general wards	NR	NR	NR	Phenotypic
Ayzanoa (2025) [22]	Peru	2017–2019	Cross-sectional genomic surveillance	1	Pediatric inpatient wards	Yes	No	Both	Phenotypic
Alvarez (2006) [78]	Colombia	2001–2003	Multicenter surveillance, observational	14	ICU (adult, pediatric, neonatal, burn unit, surgical, medical, cardiovascular)	Yes	NR	Both	Phenotypic
Calva Delgado (2016) [34]	Ecuador	2013	Cross-sectional laboratory surveillance	2	Hospital wards (specific wards NR)	NR	NR	NR	Both
Brito (2022) [47]	Chile	2010–2013	Cross-sectional molecular epidemiology	2	Multiple wards (ICU, surgical wards, etc.)	NR	Yes	Infection	Genotypic
Wozniak (2012) [33]	Chile	2006–2011	Cross-sectional, laboratory-based, multicenter surveillance	2	Hospital wards (specific wards NR)	NR	NR	Infection	Both
Velandia (2018) [79]	Colombia	2014–2014	Cross-sectional	1	Hospital wards (specific wards NR)	NR	Yes	Both	NR
Sennati (2012) [80]	Argentina	2010	Cross-sectional	15	Hospital wards (specific wards NR)	Yes	Yes	Both	Phenotypic
Rincon Cruz (2013) [49]	Argentina	2010	Multicenter point-prevalence surveillance	15	Hospital wards (specific wards NR)	NR	Yes	Infection	Both
Scheffer (2010) [81]	Brazil	2003–2005	Cross-sectional surveillance	1	ICU, internal medicine units, surgical units, emergency, other inpatient units	NR	No	NR	Phenotypic
Cuicapuza (2024) [82]	Peru	2018	Cross-sectional laboratory-based	1	Hospital wards (specific wards NR)	NR	Yes	Infection	Genotypic
Martínez (2012) [83]	Colombia	2005–2007	Cross-sectional surveillance	1	ICU, NICU	Yes	No	Infection	Genotypic
Echegorry (2024) [27]	Argentina	2021	Prospective, open, multicenter prevalence	182	Critical and non-critical areas	NR	NR	Infection	Genotypic
Faccone (2023) [24]	Argentina	2020–2021	Cross-sectional molecular surveillance	28	Multiple wards (ICU, medical/surgical wards, etc.)	Yes	No	Both	Genotypic
Picão (2012) [84]	Brazil	2003	Case series	1	Surgical ward, urology ward	No	No	Infection	Genotypic

Footnotes: Abbreviations. AmpC: AmpC β-lactamase; ICU: intensive care unit; NICU: neonatal intensive care unit; PICU: pediatric intensive care unit; ER: emergency room; CCU: coronary care unit;); NR: not reported in the source; PCR: polymerase chain reaction. Notes. Publication year appears in parentheses after the first author’s surname (e.g., *Favier (2021)*). “Data collection period” refers to the years during which specimens or surveillance data were obtained. “No. of centers (n)” counts distinct hospitals/clinics contributing isolates; when the exact number was not provided but clearly >1, we use “>1 (exact n NR)”. “Care setting(s)” lists the clinical areas from which isolates originated, commas separate settings. “Pediatric patients included” and “Outpatients included” indicate whether these populations were part of the sampling frame. “Case type (colonization vs. infection)” classifies isolates as derived from clinical infections, colonization/screening, or both, per the original authors; Both indicates a mixture within the study. “AmpC test type” records whether AmpC was assessed by phenotypic methods (e.g., inhibitor-based/cefoxitin screening, disk or MIC approaches) and/or by genotypic methods (e.g., PCR/sequencing); details vary by study and are not harmonized here. Terminology, spelling, and punctuation have been standardized across rows (in dashes in ranges; consistent Yes/No/NR). Where diacritics differed across sources, we retained the most common form; please verify against the original citations before production.

**Table 2:** Pooled prevalence of AmpC β-lactamase-producing isolates in South American hospitals by bacterial group.

Bacterial Group	Pooled Prevalence % (95% CI)	95% PI	n Isolates (k)	Heterogeneity (τ^2^ [logit], I^2^ %, Q, *p*)	GRADE Quality (Reasons)
Overall (all microorganisms)	11.7% (11.4–12.0 %)	11.4–12.0%	48 801 (69)	τ^2^ = 0; I^2^ = 97.2%; Q = 2 429.55; *p* < 0.001	Low—risk of bias, high heterogeneity, potential publication bias
*Enterobacter* spp.	46.0% (43.7–48.4 %)	43.6–48.5%	1 684 (30)	τ^2^ = 0; I^2^ = 30.9%; Q = 41.96; *p* = 0.057	Moderate—some risk of bias, moderate heterogeneity
*Salmonella* spp.	28.5% (21.2–37.0 %)	19.6–39.3%	123 (7)	τ^2^ = 0; I^2^ = 0.0%; Q ≈ 0; *p* = 1.000	Moderate—imprecision due to few studies; some risk of bias
*Morganella* spp.	20.2% (14.8–27.0 %)	14.5–27.6%	168 (18)	τ^2^ = 0; I^2^ = 0.0%; Q = 1.01; *p* = 1.000	Low—imprecision and potential study bias
*Serratia* spp.	20.0% (17.2–23.0 %)	17.1–23.2%	736 (21)	τ^2^ = 0; I^2^ = 0.0%; Q = 10.30; *p* = 0.962	Moderate—moderate evidence, low heterogeneity
*Pseudomonas* spp. †	19.3% (18.0–20.7 %)	18.0–20.7%	3 434 (28)	τ^2^ = 0; I^2^ = 83.4%; Q = 162.31; *p* < 0.001	Low—high heterogeneity and possible publication bias
*Citrobacter* spp.	18.9% (14.0–25.2 %)	13.7–25.6%	190 (19)	τ^2^ = 0; I^2^ = 0.0%; Q = 10.96; *p* = 0.896	Low—imprecision, moderate risk of bias
*Acinetobacter* spp. †	16.7% (14.8–18.7 %)	14.6–19.0%	1 410 (12)	τ^2^ = 0; I^2^ = 0.0%; Q = 1.06; *p* = 1.000	Moderate—limited number of studies, mild small-study bias
*Klebsiella* spp.	7.05% (6.60–7.53 %)	6.6–7.6%	11 583 (42)	τ^2^ = 0; I^2^ = 78.7%; Q = 192.15; *p* < 0.001	Low—high heterogeneity, possible small-study bias
*Proteus* spp.	5.23% (3.87–7.03 %)	3.8–7.1%	784 (27)	τ^2^ = 0; I^2^ = 0.0%; Q = 19.87; *p* = 0.798	Low—imprecision and few events
*Escherichia* spp.	4.48% (4.03–4.98 %)	4.0–5.0%	7 388 (39)	τ^2^ = 0; I^2^ = 93.4%; Q = 575.38; *p* < 0.001	Low—very high heterogeneity and possible small-study bias

Footnote: Values represent pooled prevalences of AmpC β-lactamase-producing isolates in each genus (number of AmpC-positive isolates/total isolates) × 100. Genus names are italicized; “spp.” is not. † indicates taxonomic membership of non-Enterobacterales label. n = number of isolates; k = number of studies. Pooled prevalences were estimated using a random-effects generalized linear mixed model with a logit transformation and maximum-likelihood estimator for τ^2^. Binomial 95% confidence intervals (CIs) were obtained by back-transforming the logit CIs. τ^2^ values are reported on the logit scale. Heterogeneity statistics include τ^2^, inconsistency (I^2^), Cochran’s Q (with corresponding *p* values), and 95% prediction intervals (PIs); we present PIs for all main estimates (not only those with I^2^ ≥ 75%). Egger’s test (intercept ≈ 0) was performed on the logit scale; *p* values < 0.10 suggest potential small-study bias. GRADE quality of evidence is categorized as Very low, Low, Moderate, or High, with reasons for downgrading being risk of bias, inconsistency (heterogeneity), imprecision (wide CIs or few studies), indirectness (limited generalizability), publication bias, or other.

## Data Availability

No new data were created or analyzed in this study.

## Supplementary Materials

The following supporting information can be downloaded at https://www.mdpi.com/article/10.3390/tropicalmed10100280/s1. Captions and legends of the Supplementary Materials: Figure S1. PRISMA 2020 flow diagram for study selection: AmpC β-lactamase prevalence in South American hospital isolates; Figure S2. Country-specific pooled prevalence of AmpC β-lactamase–producing isolates in South American hospitals (random-effects meta-analysis); Figure S3. Funnel plot and study effects for the pooled prevalence of AmpC β-lactamase–producing isolates; Figure S4. Pooled prevalence of AmpC β-lactamase–producing isolates in South American hospitals for each bacterial genus (study-level forest plots); Table S1. Univariable mixed-effects meta-regression of moderators of AmpC β-lactamase prevalence in hospital studies from South America; Table S2. Multivariable mixed-effects meta-regression of study-level moderators of AmpC β-lactamase prevalence in South American hospital studies.

## Abbreviations

The following abbreviations are used in this manuscript:

3GCRE	Third-generation cephalosporin-resistant Enterobacterales
AmpC	AmpC β-lactamase
AMR	Antimicrobial resistance
AMS	Antimicrobial stewardship
BDM	Broth dilution method
CI	Confidence interval
CLSI	Clinical and Laboratory Standards Institute
CRE	Carbapenem-resistant Enterobacterales
ESBL	Extended-spectrum β-lactamase
EUCAST	European Committee on Antimicrobial Susceptibility Testing
GLMM	Generalized Linear Mixed Model
GNB	Gram-negative bacteria
GRADE	Grading of Recommendations Assessment, Development and Evaluation
HC3	Huber–White cluster-robust standard errors type 3
I^2^	I-squared
ICU	Intensive Care Unit
ICUs	Intensive Care Units
JBI	Joanna Briggs Institute
LMICs	Low- and middle-income countries
MALDI-TOF	Matrix-assisted laser desorption/ionization-time of flight
MDR	Multidrug-resistant
MIC	Minimum inhibitory concentration
OR	Odds ratio
PCR	Polymerase chain reaction
PCR-RFLP	PCR with restriction fragment length polymorphism
PCR-SSCP	PCR with single-strand conformational polymorphism
PRISMA	Preferred Reporting Items for Systematic Reviews and Meta-Analyses
PROSPERO	International Prospective Register of Systematic Reviews
Q	Cochran’s Q statistic
QM	Omnibus test of moderators
REML	Restricted Maximum Likelihood
WHO	World Health Organization
τ^2^	Tau-squared
