# Supplementary material for: AmpC β-Lactamase-Producing Microorganisms in South American Hospitals: A Meta-Regression Analysis, Meta-Analysis, and Review of Prevalence
Source: Trop Med Infect Dis. 2025 Sep 29;10(10):280. doi: 10.3390/tropicalmed10100280 (PMC12567592; doi:10.3390/tropicalmed10100280)
Supplement: Supplementary file 1 [file tropicalmed-10-00280-s001.zip › tropicalmed-3850129-supplementary.pdf]

Tropical Medicine and Infectious Disease

MDPI

Review

AmpC β-lactamase–producing microorganisms in South American hospitals: a meta-regression analysis, meta-analysis and review of prevalence

Supplementary file

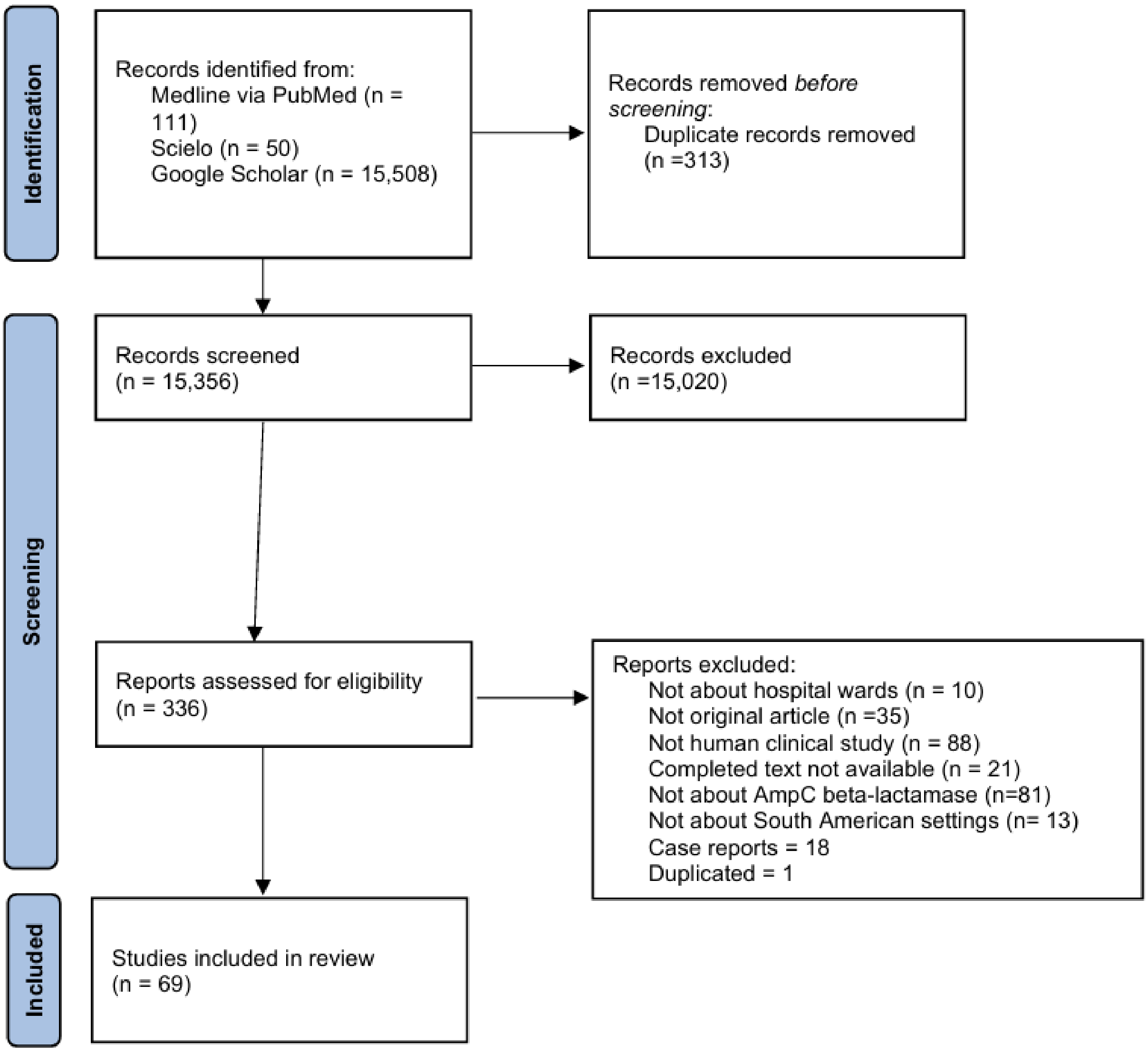

Figure S1. PRISMA 2020 flow diagram for study selection: AmpC β-lactamase prevalence in South American hospital isolates. *Footnote:* Abbreviations. PRISMA: Preferred Reporting Items for Systematic Reviews and Meta-Analyses; AmpC: AmpC β-lactamase; n: number of records/studies; MEDLINE: Medical Literature Analysis and Retrieval System Online; SciELO: Scientific Electronic Library Online. Notes. The diagram follows PRISMA 2020 guidance. Database searches covered MEDLINE via PubMed, SciELO, and Google Scholar. Titles/abstracts were screened against predefined eligibility criteria (hospital-based human clinical studies reporting AmpC prevalence in South American settings). Full-texts were sought for all potentially eligible reports. Reasons for exclusion at the eligibility stage are itemized with counts; totals equal the difference between reports assessed and studies included.

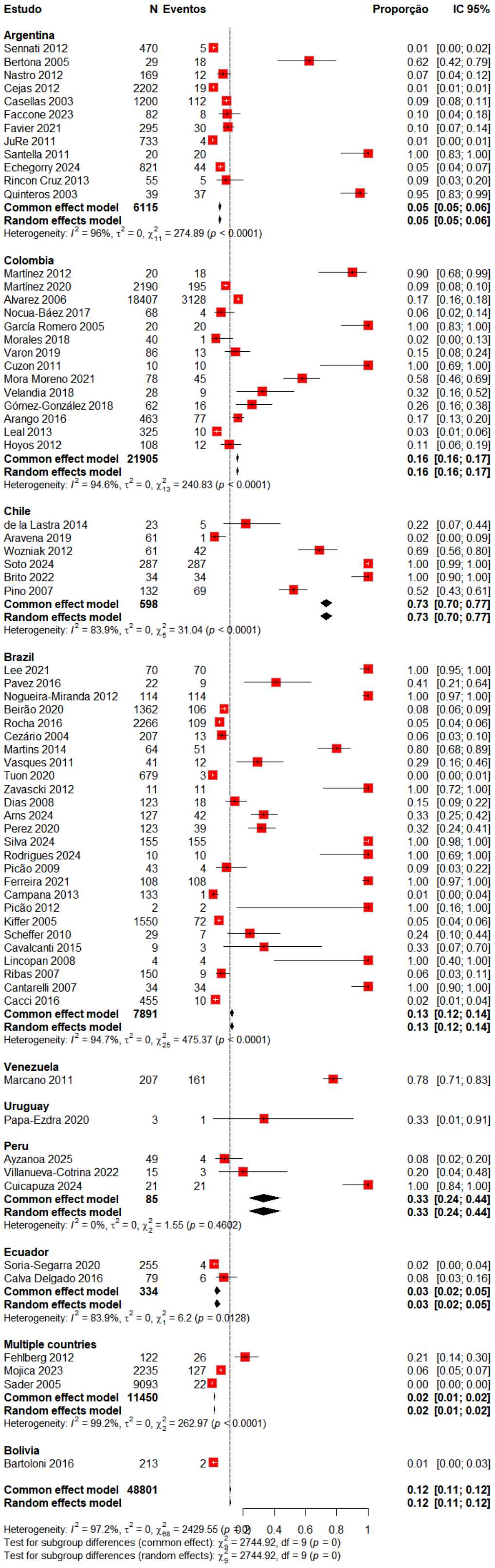

Figure S2. Country-specific pooled prevalence of AmpC β-lactamase–producing isolates in South American hospitals (random-effects meta-analysis).

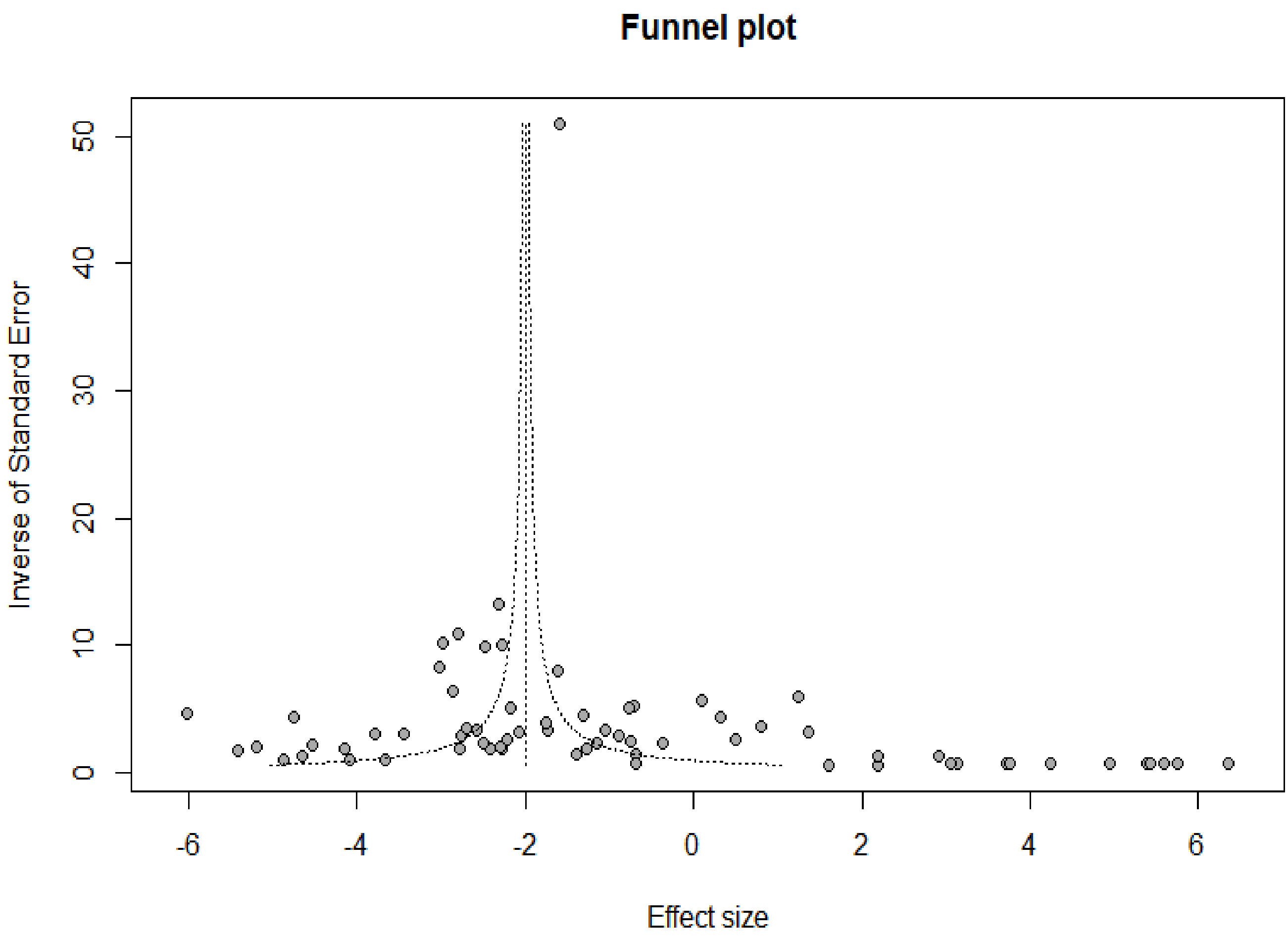

Figure S3. Funnel plot and study effects for the pooled prevalence of AmpC β-lactamase–producing isolates. Footnotes: AmpC: AmpC β-lactamase; SE: standard error; CI: confidence interval; RE: random-effects; DL/REML: DerSimonian–Laird / restricted maximum likelihood estimators; LP: logit prevalence (logit-transformed proportion); k: number of studies. Notes. Each dot is a study's logit prevalence estimate; distance from the top reflects its standard error (SE) (or precision = 1/SE if you retain that axis).

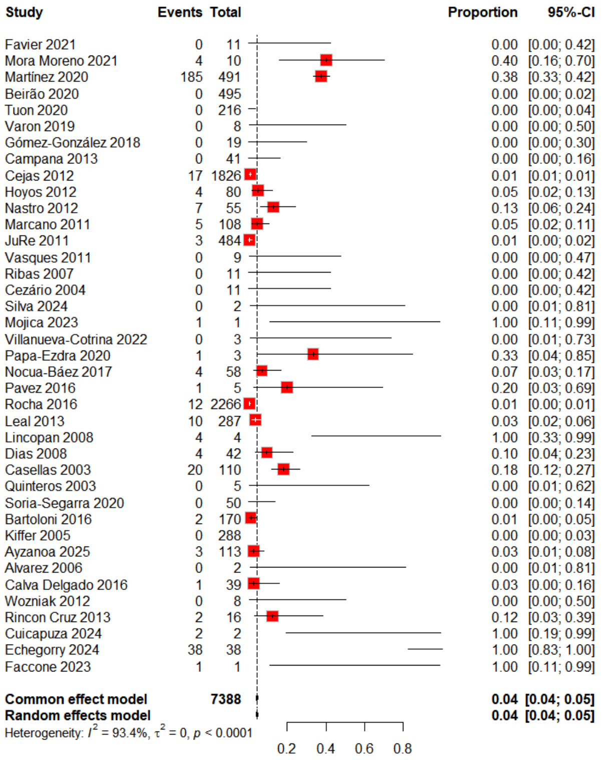

a. Escherichia spp.

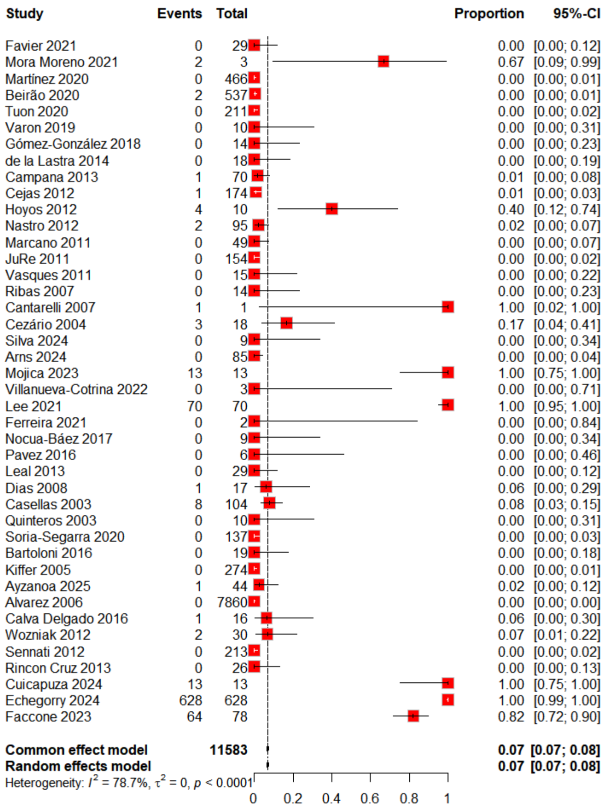

b. Klebsiella spp.

Figure S4. Pooled prevalence of AmpC β-lactamase–producing isolates in South American hospitals for each bacterial genus (study-level forest plots). Abbreviations. AmpC: AmpC β-lactamase; CI: confidence interval; RE: random-effects; FE: fixed-effect; I^2: inconsistency index; τ^2: between-study variance; k: number of studies; n: number of isolates.

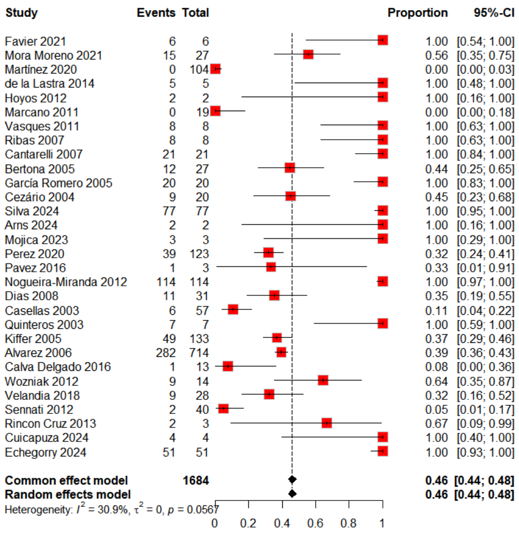

c. *Enterobacter* spp.

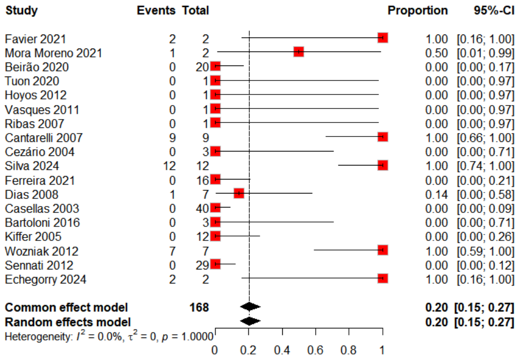

d. *Morganella* spp.

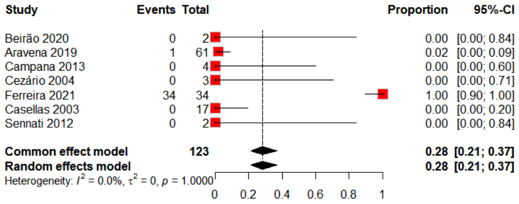

e. *Salmonella* spp.

Figure S4. (Continued) Pooled prevalence of AmpC β-lactamase–producing isolates in South American hospitals for each bacterial genus (study-level forest plots). Abbreviations. AmpC: AmpC β-lactamase; CI: confidence interval; RE: random-effects; FE: fixed-effect; I^2: inconsistency index; τ^2: between-study variance; k: number of studies; n: number of isolates.

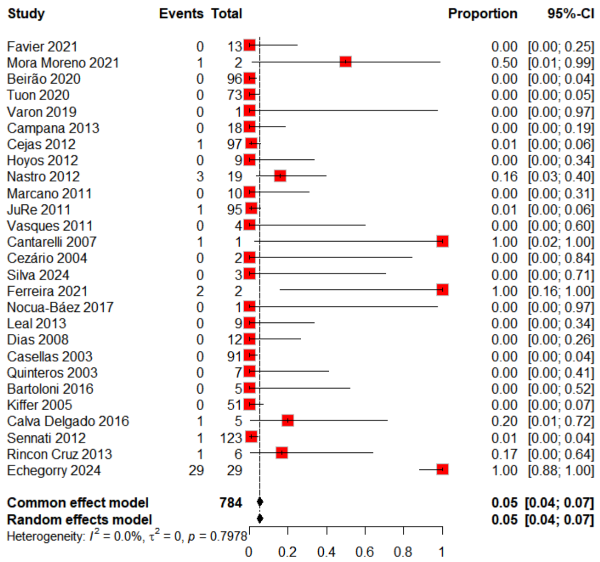

f. *Proteus* spp.

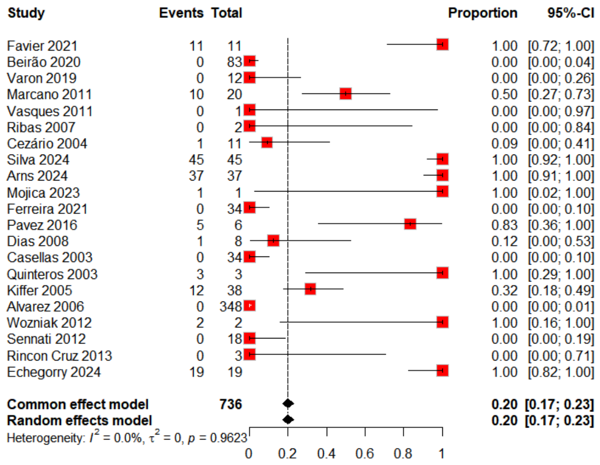

g. *Serratia* spp.

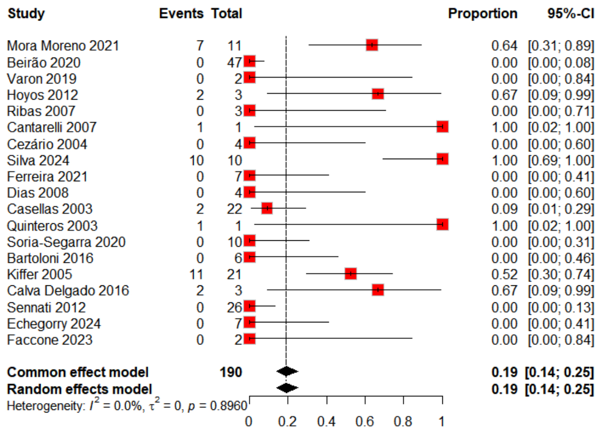

h. *Citrobacter* spp.

Figure S4. (Continued) Pooled prevalence of AmpC β-lactamase–producing isolates in South American hospitals for each bacterial genus (study-level forest plots). Abbreviations. AmpC: AmpC β-lactamase; CI: confidence interval; RE: random-effects; FE: fixed-effect; I^2: inconsistency index; τ^2: between-study variance; k: number of studies; n: number of isolates.

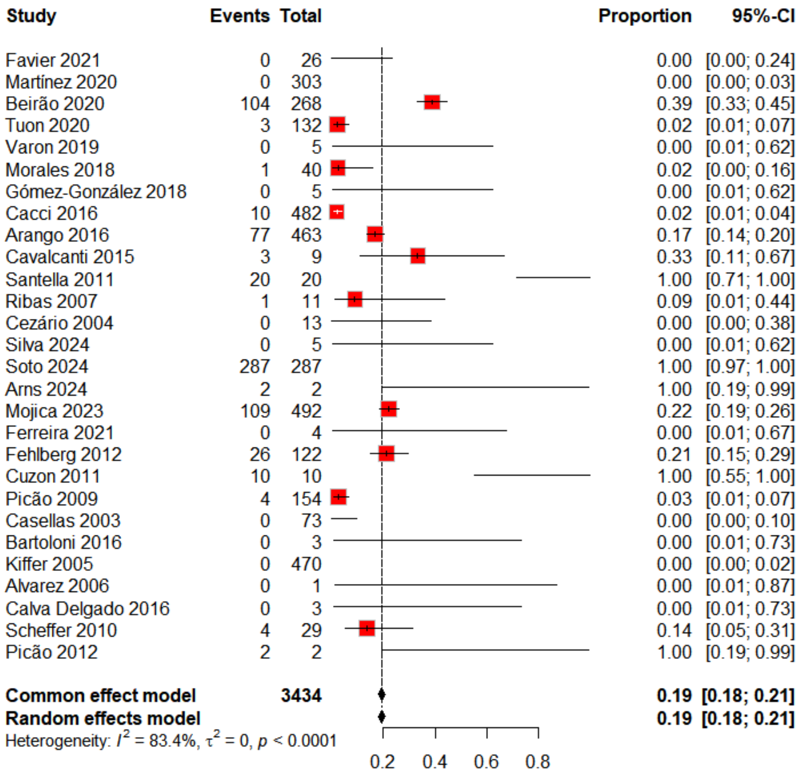

i. ***Pseudomonas*** **spp.**

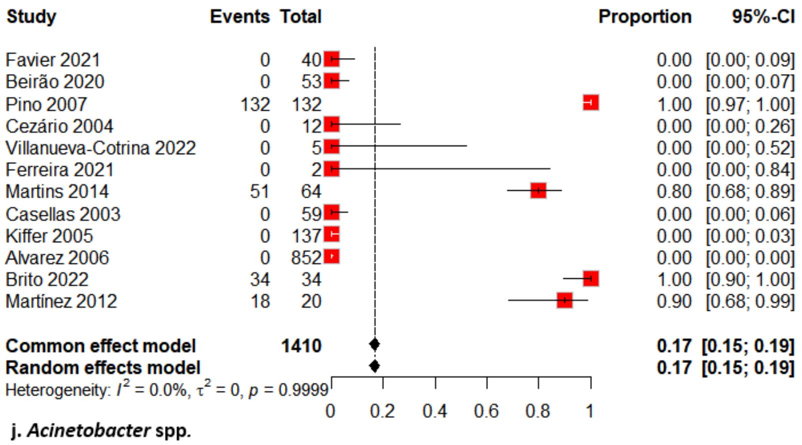

j. ***Acinetobacter*** **spp.**

Figure S4. (Continued) Pooled prevalence of AmpC β-lactamase–producing isolates in South American hospitals for each bacterial genus (study-level forest plots). Abbreviations. AmpC: AmpC β-lactamase; CI: confidence interval; RE: random-effects; FE: fixed-effect; I²: inconsistency index; τ²: between-study variance; k: number of studies; n: number of isolates.

Table S1. Univariable mixed-effects meta-regression of moderators of AmpC β-lactamase prevalence in hospital studies from South America

Moderator (reference level)	Contrast (vs ref.)	β (logit) [95% CI]	OR [95% CI]	p-value	QM (p)	Pseudo-R^2	τ^2	I^2	k
**Year of data collection (per 1 year)**	—	−0.03 [−0.14; 0.08]	0.97 [0.87; 1.08]	0.566	0.566	0.0%	81.663	99.4%	69
**Country (reference: Brazil)**	Argentina	−1.67 [−3.60; 0.26]	0.19 [0.03; 1.30]	0.090	0.061	6.6%	75.386	99.3%	69
	Chile	1.68 [−0.75; 4.10]	5.34 [0.47; 60.46]	0.176					
	Colombia	−0.70 [−2.55; 1.15]	0.50 [0.08; 3.14]	0.457					
	Other	−1.75 [−3.76; 0.25]	0.17 [0.02; 1.28]	0.086					
**AmpC test type (reference: phenotypic only)**	Both	0.39 [−1.21; 1.99]	1.47 [0.30; 7.29]	0.634	0.497	0.0%	79.151	99.5%	68
	Genotypic only	1.07 [−0.70; 2.83]	2.90 [0.50; 17.01]	0.237					
**Outpatients included (reference: No)**	Yes	−0.98 [−2.81; 0.86]	0.38 [0.06; 2.36]	0.297	0.297	0.0%	74.258	98.9%	43
**Risk of bias (JBI), per 1-point increase**	Per 1 point	−0.53 [−1.10; 0.04]	0.59 [0.33; 1.04]	0.070	0.070	4.1%	77.374	99.5%	69
**Children/adolescents included (reference: No)**	Yes	−1.77 [−3.31; −0.22]	0.17 [0.04; 0.80]	**0.025**	**0.025**	9.7%	49.052	99.3%	35
**Sample origin (reference: infection)**	Both	−0.25 [−2.13; 1.64]	0.78 [0.12; 5.14]	0.798	0.967	0.0%	86.977	99.1%	51
	Colonization	−0.17 [−6.08; 5.73]	0.84 [0.00; 308.28]	0.954					
**Focus on a specific genus (reference: specific)**	Various/any	−1.99 [−3.29; −0.68]	0.14 [0.04; 0.51]	**0.003**	**0.003**	11.1%	71.721	99.5%	69
**AmpC detection objective (reference: primary)**	Secondary	−1.42 [−3.00; 0.16]	0.24 [0.05; 1.17]	0.078	0.078	2.3%	78.817	99.5%	69

Footnote: AmpC: AmpC β-lactamase (enzyme that can inactivate many β-lactam antibiotics); β (logit): regression coefficient estimated on the logit-transformed prevalence; OR: odds ratio = exp(β), interpreted as the change in odds of AmpC positivity for the stated contrast (for continuous moderators, OR is per one-unit increase); 95% CI: 95% confidence interval; QM (p): omnibus p-value for the moderator from the mixed-effects meta-regression; Pseudo-R^2: proportional reduction in τ^2 versus the intercept-only model; τ^2: between-study variance; I^2: proportion of total variability due to heterogeneity; k: number of included effect sizes/studies for the moderator. Phenotypic testing refers to biochemical/phenotypic detection methods; Genotypic to molecular detection of AmpC genes; Both indicates studies using both methods. Sample origin—Other includes non-infection sources (e.g., colonization/surveillance). Focus on a specific group means studies restricted to a particular bacterial genus or species; Various/any means broader, mixed groups. Coefficients and p-values are from univariable meta-regressions; OR 95% CIs were computed by exponentiating the reported β 95% CIs. Bold p-values indicate $p < 0.05$.

Table S2. Multivariable mixed-effects meta-regression of study-level moderators of AmpC β-lactamase prevalence in South American hospital studies

Moderator (reference level)	Contrast (vs ref.)	OR [95% CI]	β (logit) [95% CI]	p-value	Pseudo-R^2 global	τ^2 (médio)	I^2 (médio)	QM (p)	k
**Children/adolescents included (reference: No)**	Yes	0.17 [0.03; 1.16]	−1.74 [−3.64; 0.15]	0.069	**19.5%**	62.613	99.3%	19.143 (**0.002**)	69
**Risk of bias (JBI), per 1-point increase**	Per 1 point	0.96 [0.50; 1.85]	−0.04 [−0.70; 0.62]	0.901					
**Focus on a specific genus (reference: specific)**	Various/any	0.14 [0.04; 0.57]	−1.96 [−3.35; −0.57]	**0.007**					

Footnote: AmpC: AmpC β-lactamase (an enzyme that inactivates many β-lactam antibiotics); β (logit): regression coefficient on the logit-transformed prevalence; OR: odds ratio = exp(β), interpreted as the multiplicative change in the odds of AmpC positivity for the stated contrast (for continuous moderators, per one-unit increase); 95% CI: 95% confidence interval; p-value: two-sided significance level; JBI: Joanna Briggs Institute; Risk of bias (JBI): summary JBI score, analyzed per 1-point increase; Pseudo-R^2 (overall): proportional reduction in residual between-study variance (τ^2) relative to the intercept-only model; τ^2 (residual): estimated between-study variance on the logit scale after accounting for moderators; I^2 (residual): proportion of total variability due to heterogeneity remaining after moderators; QM (p): omnibus Wald test statistic for the full multivariable model with its p-value; k: number of included effect sizes/studies. "Specific group" indicates studies focused on a single bacterial genus or species; "Various/any" indicates broader.

Supplementary information

Supplementary Note 1: Google Scholar search strategy:

"south america" | brazil | argentina | colombia | chile | suriname | ecuador | guyana | paraguay | uruguay | venezuela | bolivia | peru hospital | emergency | icu | "intensive care" ampc

Supplementary Note 2: Scielo search strategy:

"AmpC" – just one term to get highest sensitivity search results.
